# Mental health care for youth with rheumatologic diseases – bridging the gap

**DOI:** 10.1186/s12969-017-0214-9

**Published:** 2017-12-28

**Authors:** Alaina M. Davis, Tamar B. Rubinstein, Martha Rodriguez, Andrea M. Knight

**Affiliations:** 1 Division of Pediatric Rheumatology, Vanderbilt University Medical Center, Monroe Carell Junior Children’s Hospital at Vanderbilt, 2200 Children’s Way, Doctor’s Office Tower 11240, Nashville, TN 37232 USA; 2 Division of Pediatric Rheumatology, Albert Einstein College of Medicine, Children’s Hospital at Montefiore/ Albert Einstein College of Medicine, 3415 Bainbridge Avenue, Bronx, NY 10467 USA; 3 Section of Pediatric Rheumatology, Riley Hospital for Children at Indiana University Health, 705 Riley Hospital Dr, Indianapolis, IN 46202 USA; 4 Division of Rheumatology, The Children’s Hospital of Philadelphia, Roberts Center for Pediatric Research, 2716 South St, Ste 10253, Philadelphia, PA 19146 USA; 5 The Children’s Hospital of Philadelphia, Roberts Center for Pediatric Research, Center for Pediatric Clinical Effectiveness, 2716 South St, Ste 10253, Philadelphia, PA 19146 USA; 6 The Children’s Hospital of Philadelphia, Roberts Center for Pediatric Research, PolicyLab, 2716 South St, Ste 10253, Philadelphia, PA 19146 USA

**Keywords:** Pediatrics, Rheumatologic diseases, Mental health, Medical education

## Abstract

Youth with rheumatologic diseases have a high prevalence of comorbid mental health disorders. Individuals with comorbid mental health disorders are at increased risk for adverse outcomes related to mental health as well as their underlying rheumatologic disease. Early identification and treatment of mental health disorders has been shown to improve outcomes, but current systems of care fall short in providing adequate mental health services to those in need. Pediatric rheumatologists are uniquely positioned to provide mental health screening and intervention for youth with rheumatologic diseases due to the frequency of patient encounters and ongoing therapeutic relationship with patients and families. However, additional training is likely required for pediatric rheumatologists to provide effective mental health care, and focusing efforts on providing trainees with mental health education is key to building competency. Potential opportunities for improved mental health education include development of clinical guidelines regarding mental health screening and management within pediatric rheumatology settings and incorporation of mental health didactics, workshops, and interdisciplinary clinic experiences into pediatric rheumatology fellowship curricula. Additional steps include mental health education for patients and families and focus on system change, targeting integration of medical and mental health care. Research is needed to better define the scope of the problem, determine effective strategies for equipping pediatric rheumatologists with skills in mental health intervention, and develop and implement sustainable systems for delivery of optimal mental health care to youth with rheumatologic diseases.

## Background

Beyond the long-established detrimental impact that chronic disease has on an individual’s mental health, we now appreciate that the relationship between chronic physical disease and mental illness is bidirectional [1]. As such, providers that care for patients with chronic diseases must consider their patients’ mental health as both an outcome of the disease they are treating and a contributor to their patients’ overall health. Understanding the relationship between mental and physical health is of utmost importance in pediatric populations, in which both poor physical and mental health outcomes can affect development and lead to long-lasting consequences. Empowering pediatric rheumatologists to effectively address mental health disorders should be seen as an integral part of optimizing the care for youth with rheumatologic diseases. This review article aims to describe the burden and impact of mental health disorders in youth with rheumatologic diseases, assess current practices, and explore opportunities to improve mental health care for this population of vulnerable children.

## Burden of mental health disorders in youth with rheumatologic diseases

Children with chronic diseases have a higher prevalence of mental illness [2–5], and youth with rheumatologic diseases are no exception. Psychological comorbidity in pediatric rheumatology has been studied most for juvenile idiopathic arthritis (JIA), systemic lupus erythematosus (SLE), and fibromyalgia. Current prevalence estimates for depression and anxiety among patients with these diseases in North America, South America, Europe, and Asia range from 15 to 65% [6–14], with suicidal ideation rates as high as 14–34% [6, 15, 16]. These prevalences are disproportionately higher than those observed in healthy controls [6, 7, 9, 13, 17], and by some measures are higher compared to children with special health care needs other chronic diseases such as diabetes and asthma [18–20]. Moreover, recent studies indicate that individuals with childhood-onset rheumatologic disease may be at higher risk for mental health disorders than individuals with adult-onset disease [21, 22].

Discrepancies in reported prevalence of mental health disorders in this population are potentially due to small sample sizes with limited generalizability and the varying use of symptoms, screeners, or diagnosis codes to define mental health disease. These limitations and the overall paucity of existing data regarding mental health comorbidities across pediatric rheumatologic diseases emphasize the need for further research to describe better the burden of mental health disorders in this patient population. Large collaborative studies that draw on diverse demographics of patients will be important in defining the scope of the problem and understanding risk factors for mental health disorders in youth with rheumatologic diseases.

## Impact of mental health disorders in youth with rheumatologic diseases

Preliminary studies show that mental health disorders adversely impact quality of life, development, and disease outcomes in youth with rheumatologic diseases. Understanding these effects can illuminate strategies to optimize overall care. In youth with JIA, depression has been associated with higher disease activity, worse pain, worse general health, greater functional disability, and overall lower health related quality of life (HRQL) scores [8, 23]. In youth with SLE, depression and anxiety have been associated with higher disease activity and increased medical services use across ambulatory and acute care settings [9, 24, 25]. However, it is unclear whether these are causal relationships. Further research to investigate these relationships and other potential adverse outcomes related to mental health disorders in pediatric rheumatologic diseases is needed in order to guide development and assessment of future targeted interventions.

While the scope of the problem of mental illness in youth with rheumatologic diseases has yet to be fully investigated, studies of other pediatric chronic diseases indicate that mental illness can lead to poor outcomes during childhood [10, 26–29] and can affect development and risk of mental illness in adulthood [30–32]. To our knowledge, no studies have assessed long-term consequences of mental illness specifically in youth with rheumatologic diseases, but studies of adults with JIA found worse mental health, lower health-related quality of life scores, and higher levels of unemployment than in healthy controls [33, 34].

The impact of mental health disorders on rheumatologic diseases has been more robustly studied in adults than in children. In adults with SLE, depression is cited as the most significant predictor of poor health-related quality of life [35, 36], and positive depression screens have been associated with elevated disease activity scores in studies from around the globe including South America and Asia [37–40]. Similarly, comorbid mental health disorders in patients with rheumatoid arthritis and psoriatic arthritis have been associated with higher disease activity scores and lower likelihood of achieving remission [41–43]. Furthermore, depression is a risk factor for non-adherence to chronic medical treatment [44, 45]; and poor adherence has been associated with increased disease activity, increased ambulatory and acute care, and overall poor prognosis in adults with SLE [46–49].

Fortunately, early recognition and treatment of mental health disorders improves outcomes. In small, randomized controlled trials cognitive behavioral therapy was found to lower levels of functional disability and reported depressive symptoms in adolescents with fibromyalgia [50] and improve quality of life and adaptive pain cognitions in children with JIA [51]. Recent systematic reviews of psychological interventions for pediatric chronic illness describe programs implemented within various settings (i.e. community, school, and clinical practices) that have resulted in consistent improvement of a variety of clinical outcomes, particularly when subthreshold symptoms are recognized and managed [52–54]. In adults, early identification of depression, including subthreshold symptoms, decreases the risk for major depression and suicide [55, 56], improves social function, increases productivity, and decreases absenteeism [57, 58]. Specifically, cognitive-behavioral intervention in adults with SLE has led to significant reduction in the level of depression and anxiety compared to controls and an overall improvement in quality of life scores [59].

## Mental health care gap for youth with rheumatologic diseases

Given the impact of mental health disorders on health outcomes and the effectiveness of psychological interventions, the United States Preventive Services Task Force and the Canadian Network for Mood and Anxiety Treatment recommend screening adolescents for depression [60, 61]. The resulting American Academy of Pediatrics (AAP) Recommendations for Preventive Pediatric Health Care and the Guidelines for Adolescent Depression in Primary Care developed from a collaborate initiative in the United States (US) and Canada encourage annual depression screening for all adolescents in primary care settings [62, 63]. However, less than 1% of clinic visits included depression screening in a sample from ambulatory pediatric settings across the US between 2004 and 2013 [64]. Furthermore, in a recent survey by the AAP nearly 40% of the 512 responding pediatricians reported they lacked confidence in their ability to recognize mental health problems and greater than 50% reported they lacked confidence in their ability to treat them. These factors likely contributed to the responses from 44% of those surveyed who reported they were not interested in treating, managing, or co-managing childhood mental health disorders [65].

This situation might be acceptable if access to pediatric mental health providers were not exceedingly limited. Workforce data from the American Academy of Child and Adolescent Psychiatry indicates that there are approximately 8300 practicing child and adolescent psychiatrists in the US with over 15 million youth in need of one [66]. Additional data describes that 96% of counties in the US have an unmet need for prescribing mental health professionals [67], with an uneven distribution leaving rural and low-income populations underserved [68]. Similar data has been reported by the Child and Adolescent Mental Health in Europe project that summarized collective experiences from 15 countries within the European Union and highlighted specific gaps in provider education and available preventative services [69].

Thus, the current systems of care fall short in terms of meeting the mental health needs for youth with rheumatologic diseases. In a survey by the Childhood Arthritis and Rheumatology Research Alliance (CARRA), which includes pediatric rheumatologists from both US and Canada, 52% of surveyed members reported inadequate symptom identification; and 45% reported inadequate treatment of depression and anxiety in adolescents with SLE, identifying limited staff resources and availability of mental health providers as top barriers to mental health intervention [70]. These barriers are not exclusive to pediatric rheumatology settings and have been cited as barriers to identification and management of psychosocial problems within general pediatrics settings as well [65]. Yet, it is notable that only 31% of the 100 CARRA pediatric rheumatology centers report having an affiliated social worker or psychologist. When these providers were surveyed, 35% reported inadequate connection of patients to mental health services; and 52% reported inadequate follow-up of mental health referrals at their practice [71].

## Value of pediatric rheumatologists for identifying and addressing mental health disorders

The increased risk of mental health disorders in youth with rheumatologic diseases and their impact on clinical and psychosocial outcomes, as well as the shortage of mental health providers, underscore the need for pediatric rheumatologists to be proficient in addressing mental health needs for these vulnerable patients. Pediatric rheumatologists are optimally positioned to improve mental health intervention due to the frequency and intensity of their patient-provider interactions. Youth with SLE and MCTD have on average twice the number of rheumatology visits compared to primary care visits, and one study found youth with SLE and depressive symptoms to have fewer primary care visits than youth with SLE and no depressive symptoms [6]. Furthermore, in a qualitative interview study, almost all parents of youth with SLE and MCTD said they would feel comfortable bringing concerns about their child’s mental health to the rheumatologist; reasons for this included trust and confidence in the therapeutic relationship with his/her rheumatologist, whom they viewed as the primary doctor for their child [72]. In fact, the majority of patients surveyed in an ongoing mood screening project for youth with SLE believe that emotional health should be addressed by their rheumatologist [Rubinstein, unpublished data]. Additionally, a preliminary study of cognitive-behavioral therapy to improve pain and well-being in children with JIA indicated that this intervention within the pediatric rheumatology setting was acceptable to patients and families [51].

## Current state of mental health education for pediatric rheumatologists

To begin closing the mental health care gap for youth with rheumatologic diseases, pediatric rheumatologists must feel adept in the screening and initial management of mental health disorders. Symptoms of mental health disorders are often subtle and can be easily overlooked despite multiple interactions between youth with chronic conditions and the health care system. Thus, pediatric subspecialists need to learn how to implement formal screening methods into routine clinical care, effectively communicate with patients, families, and other providers regarding mental health, and assist with establishing and sustaining appropriate mental health intervention.

Current training program requirements outlined by the Accreditation Council of Graduate Medical Education simply call for adequate resources for an education in mental health and social services [73]. The American Board of Pediatrics (ABP) provides slightly more detailed expectations in subspecialty content outlines used to develop questions for board certification examinations. For pediatric rheumatology, the ABP lists the following three learning objectives relevant to mental health care: recognize the cardinal features of depression, determine when depression requires professional consultation, and recognize suicide warning signs [74]. The Objectives of Training in the Subspecialty of Adult and Pediatric Rheumatology from the Royal College of Physicians and Surgeons of Canada and the European Union of Medical Specialists Training Requirements for the Specialty of Rheumatology do not specifically address training for management of mental health comorbidities [75, 76].

Therefore, current training programs likely insufficiently prepare pediatric rheumatologists to manage mental health comorbidities in youth with rheumatologic disease. In a CARRA survey of 130 pediatric rheumatologists, two-thirds reported formal mental health training only in medical school and nearly 10% reported no training at all. Importantly, the respondents with higher levels of mental health training perceived a lower frequency of barriers to mental health screening, and greater than 90% of respondents expressed interest in further mental health training [70]. These data highlight the potential value that mental health education for pediatric rheumatologists may have on improving care for youth with rheumatologic diseases.

## Opportunities for improved mental health education for pediatric rheumatologists

The most basic measure to tackle this educational need might be to provide guidelines for mental health screening to pediatric rheumatologists. The American Diabetes Association and the American College of Gastroenterology both issued 2017 guidelines for standards of medical care in diabetes and preventive care in inflammatory bowel disease, respectively, that include recommendations for routine depression and anxiety screening [77, 78]. Such guidelines, however, do not exist for rheumatology. While 85% of surveyed CARRA-affiliated pediatric rheumatologists support routine screening for depression and anxiety in adolescents with SLE, only 2% of practices report routine screening with a standardized instrument [70]. Several standardized and validated instruments exist for mental health screening in children and adolescents, and many of these instruments are available free of charge and with translations in multiple languages [79, 80]. Setting a standard of pediatric rheumatology care inclusive of routine mental health screening, via guidelines and recommendations from professional associations in rheumatology, may be highly valuable to educate pediatric rheumatologists about mental health screening.

For pediatric rheumatology trainees, mental health education could be delivered in didactics within fellowship core curricula or offered in workshops at academic conferences. However, mastery of the skills to elicit information on the symptoms of a mental health disorder in a patient interview likely requires more intensive instruction from behavioral health specialists. Dedicated rotations in behavioral health clinics could provide this needed education if protected time for trainees and mental health providers is secured. Furthermore, training pediatric rheumatologists in the setting of interdisciplinary clinics, where rheumatology and behavioral health trainees work side by side, may offer the most comprehensive opportunity to educate providers and improve mental health care in pediatric rheumatology. While it potentially involves restructuring the way care is provided to patients, it has been proven to be effective for enhancing pediatric resident education in the management of behavioral health problems and improving collaboration between pediatricians and behavioral health specialists [81]. Accordingly, efforts are ongoing by the ABP to promote longitudinal partnerships between trainees and behavioral health providers within integrated ambulatory and inpatient settings [82]. These types of programs are also viewed by physicians and patients as a valuable strategy to improve access to mental health services and ease care coordination for at risk populations [83], and the approach is endorsed in a 2016 perspectives paper from the National Academy of Medicine [84].

Further research is needed to develop effective education programs that will optimize mental health care for youth with rheumatologic diseases. The National Academy of Medicine has outlined a research agenda to support an integrative mental health training paradigm with attention to the following topics: comparative studies of different structural configurations of the interface of pediatric medical care and behavioral health support; development of quality indicators that assess competencies for integrative team training and that lead to improved health outcomes for children; and study of new integrative training models for children with chronic disorders [84]. Studies investigating these topics will help determine best training practices that lead to the greatest impact on mental health for youth with rheumatologic diseases.

## Additional steps for improved mental health care for youth with rheumatologic diseases

Early and ongoing mental health education for patients and families of youth with rheumatologic diseases is essential to help increase mental health awareness and normalize attention to mental health as part of the overall care of youth with rheumatologic diseases. Normalization of mental health care is critical in reducing stigma and fear, both of which have been reported by youth with SLE and their parents as common barriers to seeking mental health treatment [72]. Partnerships with community providers and foundations can enhance dissemination of educational information and normalize mental health issues. For example, the National Resource Center on Lupus sponsored by Lupus Foundation of America publishes online educational information to help patients and families recognize warning signs of mental health disorders and find assistance for management of these disorders [85].

Increasing the availability of peer support groups and networks also has the potential to improve mental health education of youth with rheumatologic diseases. Specifically, the iPeer2Peer Program, a novel online peer mentoring program for adolescents with JIA developed by investigators in Canada, improved participants’ perceived ability to manage their disease compared to controls [86]. Similarly, Teens Taking Charge, an internet-based self-management program including disease-specific information and social support for youth with JIA in Canada, led to higher levels of knowledge and lower pain reports [87]. Improving support for families of youth with rheumatologic diseases also may positively impact patient outcomes, as higher caregiver psychological distress has been associated with worse HRQL in patients with JIA [88, 89].

While improved mental health education for both pediatric rheumatologists and patients and their families is necessary, education alone is insufficient. Sustainable systems for the delivery of mental health services must be developed. Dedicated application of implementation science principles and quality improvement methodology has been successfully utilized to improve mental health care for youth with other chronic diseases [90]. Funding is needed to support implementation research and quality improvement initiatives aimed at optimizing mental health care for youth with rheumatologic diseases. Required funding for overall increased mental health resources for this population should not be overlooked either, particularly given reported lack of time and adequate staffing for mental health screening and treatment in pediatric rheumatology settings [70]. The necessity of this funding priority aligns with the goals and expectations of our patients, as indicated in a mixed methods study of youth with SLE showing that research focused on alleviating poor psychological outcomes is viewed as a top priority in this population [91].

## Conclusions

The prevalence and burden of mental health disorders in youth with rheumatologic diseases is high. The evidence from pediatric and adult literature of rheumatologic diseases indicates that mental illness has a pervasive impact on multiple areas of health behaviors, disease outcomes, and quality of life. Improved identification and early treatment of mental health disorders may improve outcomes in this population, and pediatric rheumatologists are well situated to provide mental health screening and offer early intervention. Additional training for pediatric rheumatologists is necessary to enable provision of effective mental health care. Potential educational strategies include the development of clinical guidelines for mental health screening within pediatric rheumatology settings and revision of pediatric rheumatology fellowship cuuricula to include mental health didactics, workshops, and clinical experience in behavioral health. Improved education of patients and families is also essential in order to increase awareness of and normalize inclusion of mental health as part of overall care. Future work should focus on developing and implementing sustainable systems for the delivery of mental health care alongside medical care for youth with rheumatologic diseases. Increased advocacy for mental health resources and research funding is needed to support the above initiatives.

## Abbreviations

AAP: American Academy of Pediatrics
ABP: American Board of Pediatrics
CARRA: Childhood Arthritis and Rheumatology Research Alliance
HRQL: health related quality of life
JIA: juvenile idiopathic arthritis
MCTD: mixed connective tissue disease
SLE: systemic lupus erythematosus
US: United States
